# Relationship between meteorological and environmental factors and acute exacerbation for pediatric bronchial asthma: Comparative study before and after COVID-19 in Suzhou

**DOI:** 10.3389/fpubh.2023.1090474

**Published:** 2023-01-27

**Authors:** Suyu Guo, Dongmei Chen, Jiawei Chen, Canhong Zhu, Li Huang, Zhengrong Chen

**Affiliations:** Department of Respiratory Medicine, Children's Hospital of Soochow University, Suzhou, China

**Keywords:** bronchial asthma, epidemiology, COVID-19, meteorological, environment

## Abstract

**Objective:**

Climate and environmental change is a well-known factor causing bronchial asthma in children. After the outbreak of coronavirus disease (COVID-19), climate and environmental changes have occurred. The present study investigated the relationship between climate changes (meteorological and environmental factors) and the number of hospitalizations for pediatric bronchial asthma in Suzhou before and after the COVID-19 pandemic.

**Methods:**

From 2017 to 2021, data on daily inpatients diagnosed with bronchial asthma at Children's Hospital of Soochow University were collected. Suzhou Meteorological and Environmental Protection Bureau provided daily meteorological and environmental data. To assess the relationship between bronchial asthma-related hospitalizations and meteorological and environmental factors, partial correlation and multiple stepwise regression analyses were used. To estimate the effects of meteorological and environmental variables on the development of bronchial asthma in children, the autoregressive integrated moving average (ARIMA) model was used.

**Results:**

After the COVID-19 outbreak, both the rate of acute exacerbation of bronchial asthma and the infection rate of pathogenic respiratory syncytial virus decreased, whereas the proportion of school-aged children and the infection rate of human rhinovirus increased. After the pandemic, the incidence of an acute asthma attack was negatively correlated with monthly mean temperature and positively correlated with PM_2.5_. Stepwise regression analysis showed that monthly mean temperature and O_3_ were independent covariates (risk factors) for the rate of acute asthma exacerbations. The ARIMA (1, 0, 0) (0, 0, 0) 12 model can be used to predict temperature changes associated with bronchial asthma.

**Conclusion:**

Meteorological and environmental factors are related to bronchial asthma development in children. The influence of meteorological and environmental factors on bronchial asthma may be helpful in predicting the incidence and attack rates.

## 1. Introduction

The coronavirus disease (COVID-19) outbreak in China has been the largest outbreak of atypical pneumonia since the outbreak of severe acute respiratory syndrome coronavirus 2 (SARS-CoV-2) (1). Due to the rapid spread of COVID-19 from person to person, the Chinese government declared a national public health emergency and implemented a series of stringent prevention and control measures, including self-isolation, social isolation, community containment, traffic reduction, and a suspension of industrial and trade activities (2). COVID-19-related lockdown has improved ambient air pollution and air quality in many regions (3, 4), consequently reducing the incidence of respiratory diseases such as asthma. Epidemiological studies have proved the correlation between weather changes and environmental pollution and the outpatient visits of children with asthma (5), and confirmed that long-term exposure to air pollutants can increase the prevalence of asthma and asthma symptoms in children to varying degrees (6, 7). A study of ten European cities showed that 14% of childhood asthma flare-ups and 15% of childhood asthma exacerbations were associated with exposure to road traffic pollutants (8).

The average incidence of asthma in developing countries is 3–5%, compared to more than 20% in developed countries (9). The overall prevalence rate of childhood asthma in China from 2011 to 2018 was 3.3% (10). In recent years, the prevalence of asthma in children has increased to 4.42% (11).The purpose of this study was to investigate the relationship between meteorological and environmental factors and the number of pediatric bronchial asthma hospitalizations in Suzhou before and after the COVID-19 pandemic from 2017 to 2021 by taking advantage of changes in meteorology and environment caused by the prevention and control strategies and lockdown restrictions implemented during the COVID-19 pandemic. We can gather evidence on the effects of meteorological and environmental changes on human health and provide directions for better prevention and treatment of bronchial asthma in clinical practice by observing the potential reversibility of the effects of meteorological and environmental changes on bronchial asthma. This study focuses on bronchial asthma because it is a common respiratory disease in children and a clinical and public health priority.

## 2. Materials and methods

### 2.1 Research object

Bronchial asthma patients aged < 14 years who were hospitalized in the Respiratory Department of Children's Hospital Affiliated with Soochow University from January 1, 2017, to December 31, 2021, were included in this study. Demographic data, clinical characteristics, and laboratory test results of all pediatric patients were collected retrospectively and analyzed. The study was conducted with the approval of the Institutional Human Ethics Committee of Soochow University.

### 2.2. Surveillance of common respiratory viruses

A standard protocol was followed to collect nasopharyngeal aspirate samples from hospitalized children with bronchial asthma. Within 24 h of admission, these samples were obtained from the patients by inserting a sterile plastic catheter through the nasal cavity into the hypopharynx. Light Diagnostics Respiratory Viral Screen DFA (Chemicon International, USA, from 2001 to 2005; D^3^ Ultra^TM^ DFA Respiratory Virus Screening & ID Kit, Diagnostic Hybrids Inc., USA, from 2006 to 2011) was used to detect the respiratory syncytial virus (RSV); Influenza virus A (IV-A)and Influenza virus B (IV-B); paravienzA-1, −2, and −3; and adenovirus. Human metapneumovirus, human rhinovirus (HRV), and Mycoplasma pneumoniae (MP) were detected by real-time polymerase chain reaction (Applied Biosystems, Foster City, CA, USA). The daily total number of positive etiological cases was routinely checked and recorded, together with the number of hospitalizations for bronchial asthma.

### 2.3. Meteorological environment data

Suzhou is located in the southeast part of the Yangtze River Delta (longitude 120° E, latitude 31° N), which is a special economic zone in the marine climatic zone of the north subtropical monsoon region. Data of meteorological variables, namely, daily average temperature (°C), average relative humidity (%), total rainfall (Mm), total sunshine hours (H), and average wind speed (m/s) in Suzhou were collected from Suzhou Meteorological Bureau. Data of environmental variables, namely, PM_2.5_ (μg/m^3^), PM_10_ (μg/m^3^), NO_2_ (μg/m^3^), SO_2_ (μg/m^3^), CO (μg/m^3^), and O_3_ (μg/m^3^), were collected from Suzhou Environmental Protection Bureau. Meteorological data were obtained hourly, and daily average values were calculated. Monthly average values were calculated based on the daily average values of temperature, relative humidity, and wind speed. Total rainfall and sunshine hours were calculated as the total values for the month.

### 2.4. Statistical analysis

The percentage of samples that tested positive for respiratory pathogens was calculated on a monthly basis. We used a monthly series rather than a weekly series to reduce the amount of data. The analysis of variance was used to compare numerical data. The correlation between the incidence of acute bronchial asthma attacks and meteorological and environmental factors was assessed using Spearman's rank correlation analysis. Because of the collinearity of meteorological factors, partial correlation analysis was used to examine the relationship between meteorological and environmental factors and the incidence of acute bronchial asthma attacks. We used stepwise regression analysis after selecting the variables to examine the relationship between meteorological and environmental factors and the number of hospitalizations for asthmatic disorders.

Using the autoregressive integrated moving average (ARIMA) model, the expert modeling program in SPSS software was used to analyze multiple time series. The optimal ARIMA model is mainly used by the normalized Bayesian information criterion (BIC) value, coefficient of determination (R^2^), root mean square error (RMSE), and mean absolute percentage error (MAPE). The Ljung-Box Q test was used to assess whether the residual sequence was white noise. To examine the temporal correlation between temperature and asthma development, we fitted models with different lag structures from a month (lag 0) to ≤ 2 months (lag 2) using the distributed lag model of SPSS Expert Modeler (automatic model selection) and the customized ARIMA model. The expert modeling program automatically selected parameters such as estimator, standard deviation (standard error), *P*-value, stationary R2 value, and normalized BIC. In this case, the data from 2017 to 2019 (estimation period) were used for the multiple time series analysis based on the ARIMA model to analyze the impact of meteorological and environmental factors on bronchial asthma development. We then projected the incidence of asthmatic disorders from 2020 to 2021 (evaluation period) to assess the predictive power of the model.

All statistical tests were two-tailed, and P < 0.05 was considered to indicate statistical significance. SPSS software version 23.0 (SPSS Inc., Chicago, IL, USA), was used for all data analyses.

## 3. Results

### 3.1. Characteristics of children with bronchial asthma

Medical records of 9,322 children with acute respiratory tract infections were screened, and relevant data were collected (Table 1). Before the COVID-19 pandemic, 5,984 acute respiratory tract infection cases were identified, of which 668 (11.2%) cases were diagnosed as bronchial asthma. After the COVID-19 pandemic, 3,338 cases of acute respiratory tract infection were identified, of which 319 (9.6%) cases were diagnosed as bronchial asthma. The number of hospitalizations for bronchial asthma after the pandemic was significantly lower than before the pandemic (*P* < 0.05). Before and after the pandemic, the number of boys with bronchial asthma was 360 (53.9%) and 189 (53.2%), with an insignificant difference (*P* > 0.05). The age distribution of children with asthma ranged from 3 to 5 years of preschool age, 6–10 years of school age, and 10 to 18 years of adolescence. Current diagnostic criteria for bronchial asthma are mainly applied to older children (12). The diagnosis of asthma in children under 6 years old is still a challenging clinical problem. At present, asthma risk factor prediction models are often used in foreign countries to judge the probability of asthma in wheezing children (11). Adolescence is a transitional period for children to become adults, which has its unique physiological and psychological characteristics. Adolescent asthma has a higher incidence, mortality and poor treatment compliance, with a more pronounced disease burden compared to younger children. The characteristics of adolescence determine the difference between the management model of asthma in adolescence and that of children and adults (13, 14). So this paper is divided into pre-school age, school age and adolescence. It has been added. Children with bronchial asthma were mostly pre-school age (460 cases, 68.9%) before the pandemic and school-age (133 cases, 41.7%) after the pandemic. However, the proportion of school-age children with bronchial asthma after the pandemic was significantly lower than before the pandemic (*P* < 0.05). The results of the univariate analysis showed that the number of children with bronchial asthma who experienced shortness of breath and dyspnea (clinical symptoms) decreased after the pandemic when compared with that before the pandemic, with a significant difference (*P* < 0.05). On the contrary, no significant difference was observed in the number of children with bronchial asthma who experienced cough, fever, and wheezing (clinical symptoms) before and after the pandemic (*P* > 0.05). Overall, the incidence of bronchial asthma was highest in winter and lowest in summer. When comparing the data before and after the pandemic, the proportion of asthma-related hospitalizations increased in autumn and winter and decreased in spring and summer after the pandemic, with a significant difference (*P* < 0.05).

**Table 1:** Demographic and clinical characteristics of children hospitalized for bronchial asthma before and after the COVID-19 outbreak.

	**Before the COVID-19 pandemic**	**After the COVID-19 outbreak**	***P***
**Demographic**			
Bronchial asthma [n (%)]	668 (11.2)	319 (9.6)	0.016
**Sex**			
Male [n (%)]	360 (53.9)	189 (59.2)	0.113
Female [n (%)]	308 (46.1)	130 (40.8)	
**Age distribution**			
Preschool [n (%)]	460 (68.9)	175 (54.9)	0.000
School-age [n (%)]	160 (24.0)	133 (41.7)	
Adolescence [n (%)]	48 (7.1)	11 (3.4)	
**Clinical characteristics**			
Cough [n (%)]	649 (97.2)	312 (97.8)	0.551
Fever [n (%)]	424 (63.5)	197 (61.8)	0.601
Wheezing [n (%)]	527 (78.9)	244 (76.5)	0.393
Breathlessness [n (%)]	170 (25.4)	51 (16.0)	0.001
dyspnea [n (%)]	154 (23.1)	50 (15.7)	0.007
**Season**			
Spring [n (%)]	161 (24.1)	64 (20.1)	0.022
Summer [n (%)]	105 (15.7)	33 (10.3)	
Autumn [n (%)]	138 (20.7)	83 (26.0)	
Winter [n (%)]	264 (39.5)	139 (43.6)	

### 3.2. Etiological characteristics

**Figure 1:**
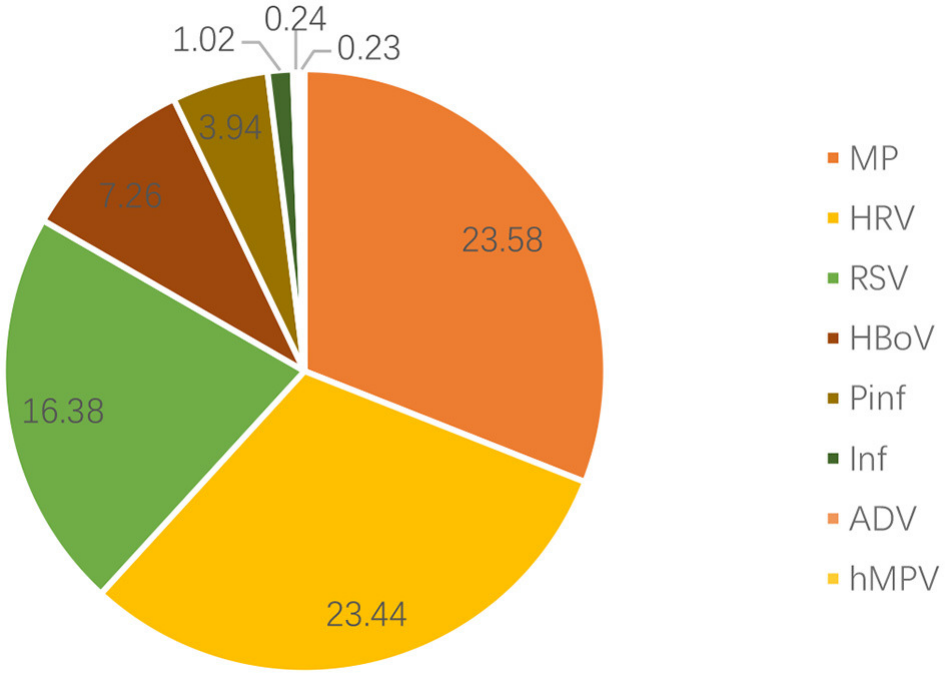
Distribution of pathogens in the nasopharyngeal samples of children with bronchial asthma. RSV, respiratory syncytial virus; HRV, human rhinovirus; MP, Mycoplasma pneumoniae; HBoV, human bocavirus; Pinf, parainfluenza virus; Inf, influenza virus; ADV, adenovirus; hMPV, human metapneumovirus.

Nasopharyngeal secretion samples of 987 children with bronchial asthma were assessed, and one or more pathogens were detected in 708 (71.7%) samples. The top three pathogens detected were MP (23.58%), HRV (23.44%), and RSV (16.38%; Figure 1). As for the seasonal distribution patterns of these three most common respiratory viruses (Table 2), RSV detection rate showed no change when comparing the data before and after the pandemic. RSV activity showed a significant peak in winter. When compared to before the pandemic, the RSV detection rate in each season decreased significantly after the pandemic. Furthermore, HRV activity peaked in autumn/winter, and HRV detection rates in summer, autumn, and winter after the pandemic were significantly higher than before. The detection rate of MP peaked in the summer/autumn, and the detection rate in the summer and autumn increased significantly after the pandemic when compared to before the pandemic. Regarding the age distribution of the three respiratory viruses (Table 3), the RSV detection rate was mainly concentrated in pre-school age after the pandemic compared to before the pandemic. The HRV detection rate was mainly concentrated in pre-school age before the pandemic and at school age after the pandemic. The detection rate of the viruses in all age groups after the pandemic was higher than before, with a significant difference. MP detection rate was concentrated in school-age before the pandemic and adolescence after the pandemic. The detection rate after the pandemic was significantly higher than before, with a significant difference.

**Table 2:** Seasonal distribution of common respiratory pathogens in children with bronchial asthma before and after the COVID-19 outbreak.

	**RSV**		**HRV**		**MP**	
	**Before the COVID-19 pandemic**	**After the COVID-19 outbreak**	**Before the COVID-19 pandemic**	**After the COVID-19 outbreak**	**Before the COVID-19 pandemic**	**After the COVID-19 outbreak**
Spring	22 (13.7%)	4 (6.3%)	14 (8.7%)	5 (7.8%)	24 (14.9%)	6 (9.4%)
Summer	4 (3.8%)	0 (0)	4 (3.8%)	6 (18.2%)	44 (41.9%)	14 (42.4%)
Autumn	18 (13.0%)	8 (9.1%)	29 (21.0%)	21 (25.3%)	32 (23.2%)	29 (34.9%)
Winter	43 (16.3%)	17 (12.2%)	47 (17.8%)	40 (28.8%)	13 (4.9%)	5 (3.6%)
*X^2^*	10.411	8.489	21.534	11.758	77.988	54.566
*P*	0.015	0.037	0.000	0.008	0.000	0.000

**Table 3:** Age distribution of common respiratory pathogens in children with bronchial asthma before and after the COVID-19 outbreak.

	**RSV**		**HRV**		**MP**	
	**Before the COVID-19 pandemic**	**After the COVID-19 outbreak**	**Before the COVID-19 pandemic**	**After the COVID-19 outbreak**	**Before the COVID-19 pandemic**	**After the COVID-19 outbreak**
Pre-school	81 (17.6%)	26 (14.9%)	75 (16.3%)	31 (17.7%)	69 (15%)	19 (10.9%)
School-age	5 (3.1%)	3 (2.3%)	16 (10%)	40 (30.1%)	42 (26.3%)	32 (24.1%)
Adolescence	1 (2.1%)	0 (0)	3 (6.3%)	1 (9.1%)	2 (4.2%)	3 (27.3%)
*X^2^*	27.448	18.598	6.519	7.892	16.671	10.235
*P*	0.000	0.000	0.038	0.019	0.000	0.006

### 3.3. Data description of meteorological and environmental factors

**Figure 2:**
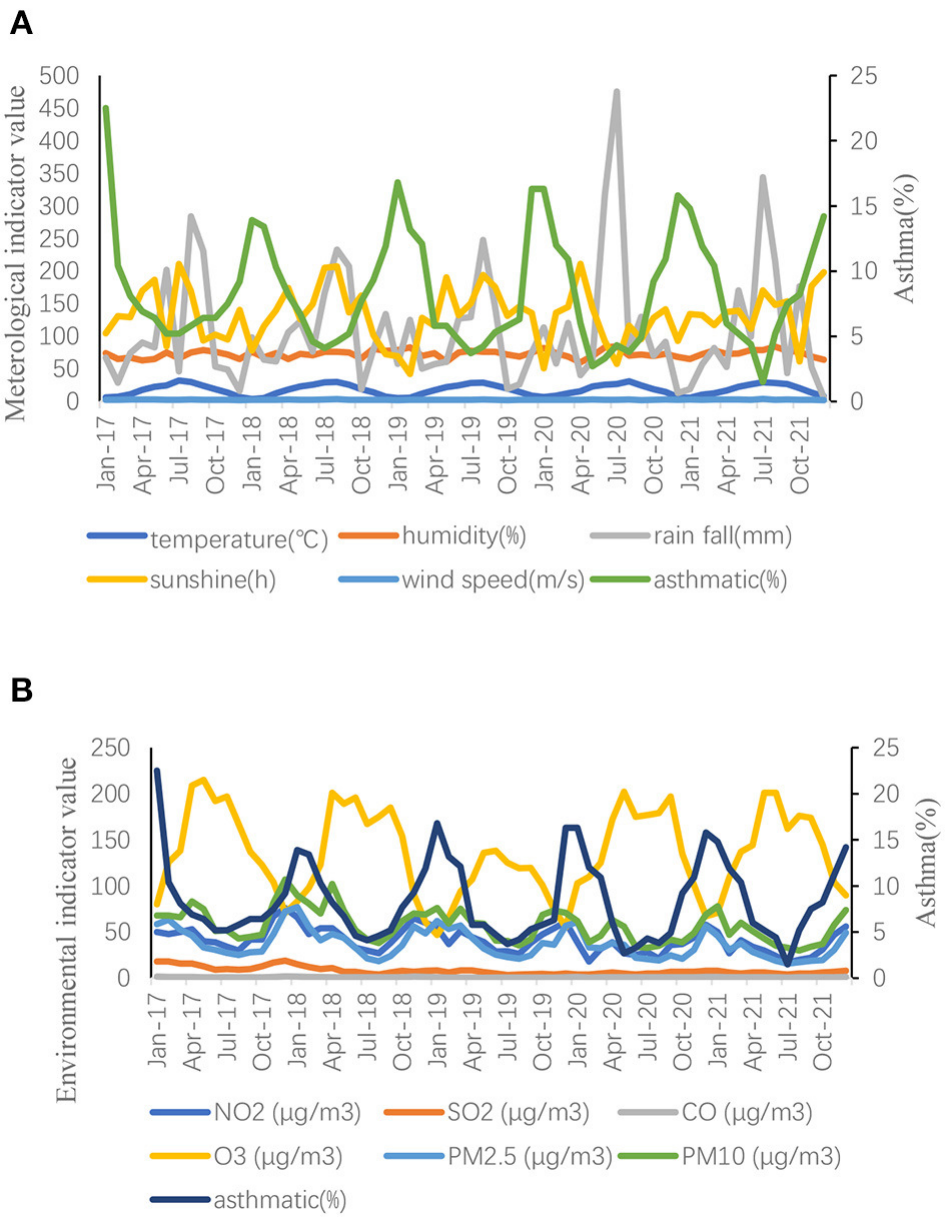
**(A, B)** Data description of meteorological and environmental variables.

Annual mean values of meteorological and environmental factors from 2017 to 2021 were recorded (Table 4). No significant difference was noted in meteorological factors when comparing the data before and after the pandemic. After the pandemic, NO_2_, SO_2_, PM_2.5_, and PM_10_ levels decreased when compared with those before the pandemic, with significant differences (*P* < 0.05). Contrarily, O_3_ levels increased after the pandemic when compared with those before the pandemic, with significant differences (*P* < 0.05). Monthly average data of meteorological and environmental variables were recorded (Figure 2).

**Table 4:** Annual mean values of meteorological and environmental variables before and after the outbreak of COVID-19.

	**Before the COVID-19 pandemic**	**After the COVID-19 outbreak**	***t***	***P***
Mean temperature (°C)	17.76 ±8.64	18.10 ±8.14	−0.155	0.877
Relative humidity (%)	72.47 ±5.45	73.62 ±6.40	−0.745	0.459
Total rainfall (mm)	139.19 ±70.64	120.27 ±14.92	0.275	0.893
Total sunshine (h)	136.01 ±42.03	128.87 ±40.41	0.568	0.687
Wind velocity (m/s)	2.57 ±0.31	2.67 ±0.39	2.424	0.059
NO_2_ (μg/m^3^)	45.91 ±12.48	33.46 ±11.59	3.895	0.000
SO2 (μg/m^3^)	9.34 ±4.59	5.75 ±1.29	4.438	0.000
CO (μg/m^3^)	0.99 ±0.38	0.96 ±0.23	0.449	0.685
O3 (μg/m^3^)	129.58 ±48.44	144.04 ±42.30	−1.191	0.039
PM2.5 (μg/m^3^)	41.43 ±15.54	30.55 ±12.45	2.866	0.006
PM10 (μg/m^3^)	63.48 ±17.84	47.79 ±14.15	3.614	0.000

### 3.4. Partial correlation analysis of meteorological and environmental factors with asthma prevalence before and after the pandemic

According to the results of partial correlation analysis (Table 5), the incidence of an acute asthma attack was negatively correlated with monthly mean temperature (*P* < 0.001, *r* = −0.755 [before the pandemic]; *P* < 0.001, *r* = −0.900 [after the pandemic]) and O_3_ (*P* < 0.05, *r* = −0.373 [before the pandemic]; *P* < 0.001, *r* = −0.804 [after the pandemic]) and positively correlated with PM_2.5_ (*P* < 0.05, *r* = 0.454 [after the pandemic]).

**Table 5:** Partial correlation analysis of meteorological and environmental factors with the incidence of bronchial asthma before and after the COVID-19 outbreak.

	**Before the COVID-19 pandemic**	**After the COVID-19 outbreak**
Mean temperature (°C)	−0.755^**^	−0.900^**^
Relative humidity (%)	0.274	0.341
Total rainfall (mm)	0.139	−0.112
Total sunshine (h)	0.270	0.289
Wind velocity (m/s)	−0.052	−0.230
NO_2_ (μg/m^3^)	−0.269	−0.021
SO2 (μg/m^3^)	−0.001	0.215
CO (μg/m3^)^	0.200	0.001
O3 (μg/m^3^)	−0.373^*^	−0.804^**^
PM2.5 (μg/m^3^)	0.454	0.576^*^
PM10 (μg/m^3^)	0.035	−0.327

*P < 0.05; **P < P 0.01.

### 3.5. Multiple stepwise regression analysis of meteorological and environmental factors and bronchial asthma development

Multiple regression analysis was performed to determine the most critical meteorological and environmental factors, taking into account the correlation between meteorological and environmental factors. Based on inflation factor variance (VIF), we excluded PM_2.5_ and PM_10_ after the pandemic and continued to perform stepwise regression analysis on the remaining variables (Table 6). The incidence of acute asthma attacks was negatively correlated with monthly mean temperature and O_3_ before and after the pandemic.

**Table 6:** Multiple stepwise regression analysis of the relationship between meteorological and environmental factors and bronchial asthma development before and after the COVID-19 pandemic.

	**Before the COVID-19 pandemic**		**After the COVID-19 outbreak**	
	**Beta**	***P***	**Beta**	***P***
Mean temperature (°C)	−0.827	0.000	−0.522	0.001
Relative humidity (%)	0.144	0.136	0.101	0.216
Total rainfall (mm)	0.174	0.135	−0.084	0.341
Total sunshine (h)	0.013	0.920	−0.114	0.106
Wind velocity (m/s)	0.048	0.652	−0.135	0.059
NO_2_ (μg/m^3^)	−0.235	0.226	−0.008	0.949
SO2 (μg/m^3^)	−0.105	0.458	0.019	0.836
CO (μg/m^3^)	0.032	0.830	0.105	0.369
O3 (μg/m^3^)	−0.293	0.026	−0.911	0.000

### 3.6. Construction of the autoregressive integrated moving average sliding model

**Figure 3:**
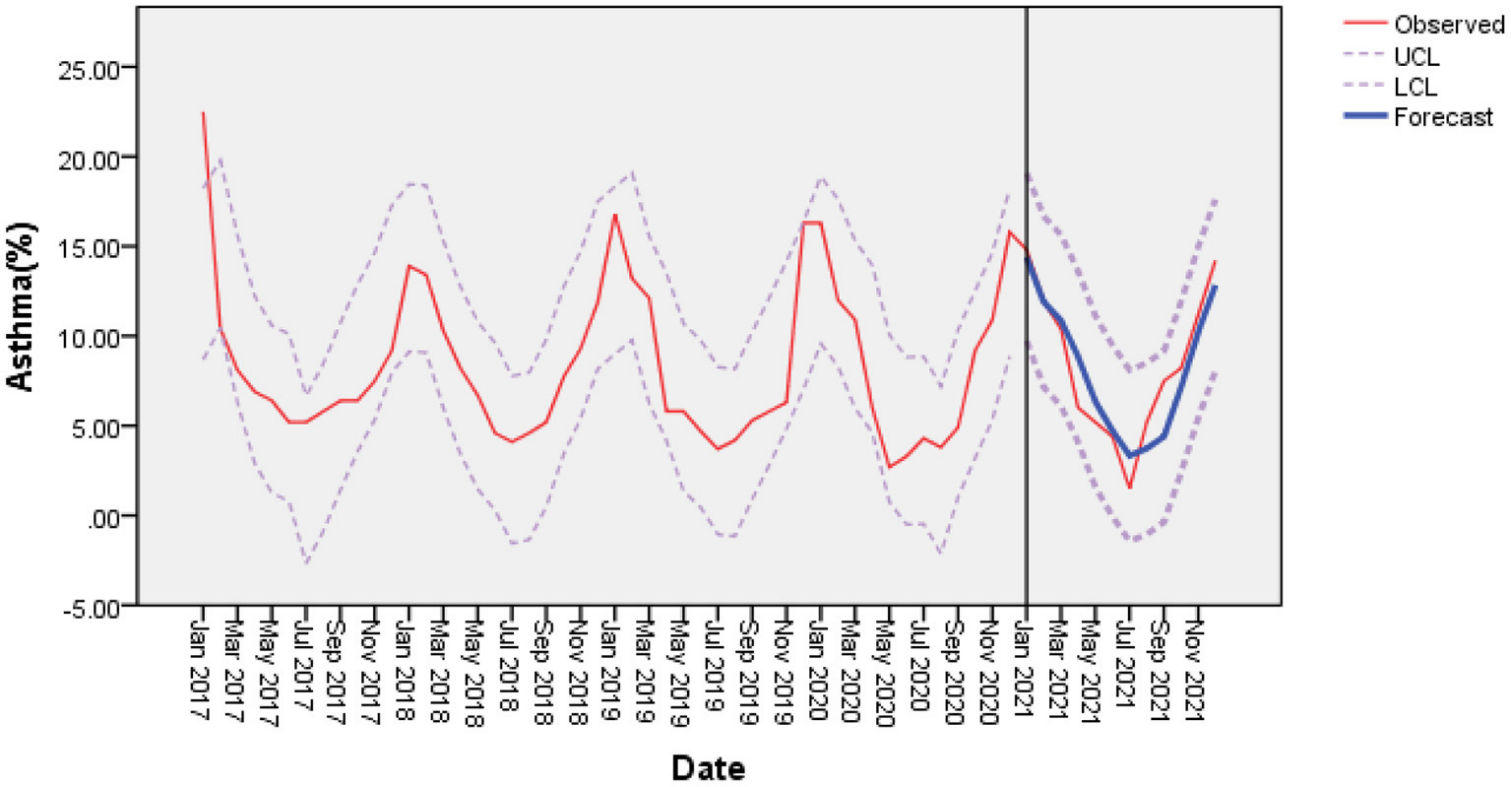
With mean temperature as a covariate, the incidence of bronchial asthma was predicted based on the ARIMA (1, 0, 0) (0, 0, 0) model. There was a good agreement between the observed and predicted incidence of bronchial asthma. LCL, lower confidence interval; UCL, upper confidence interval.

In stepwise regression analyses, monthly mean temperature and O_3_ were independent covariates significantly associated with the rate of acute asthma exacerbations. Therefore, we established a multiple time series model based on changes in temperature and O_3_ (Table 7); the results showed that R^2^ = 0.684 of the time series based on temperature was the highest. Therefore, we chose temperature for ARIMA analysis using the expert modeler (Figure 3). The ARIMA (1, 0, 0) (0, 0, 0) 12 model had the most appropriate normalized BIC (1.761); the RMSE value was 2.178, and the MAPE value was 20.582. The ARIMA model has a static R^2^ value of 0.750, which is automatically selected by the expert modeling program. In ARIMA analysis, 0 month lag temperature (β = −0.445, *t* = −10.523, *P* < 0.001) had a significant negative effect on the number of acute asthma exacerbations. The residual order was listed as white noise based on the Ljung-Box test (*P* = 0.828), which met the model evaluation criteria.

**Table 7:** Multivariate time-series model analysis for bronchial asthma from January 2017 to December 2021.

**Parameter**	**Lag time**	**Model 1: without meteorological factors**			**Model 2: with meteorological factors**			**Model 3: with environment factors**		
		**Estimate**	**S.E**.	***P***	**Estimate**	**S.E**	***P***	**Estimate**	**S.E**	***P***
MA	Lag5	0.678	0.152	0.000						
AR	Lag1				0.644	0.164	0.000			
temperature	Lag0				−0.328	0.087	0.000			
AR	Lag1							0.636	0.117	0.000
O3	Lag0							−0.065	0.013	0.000

Model 1: R-square = 0.268; Model 2: R-square = 0.684; Model 3: R-square = 0.600.

## 4. Conclusions and discussions

In China, ~4.2% of adults suffer from asthma, of which nearly 30%-50% develop from childhood (15). Environmental and genetic factors, immunological parameters, diet and nutrition, and so on are all risk factors for bronchial asthma in children (16, 17). Among these risk factors, genetic factors are the most difficult to intervene with on a large scale (18, 19). Environmental factors such as weather and indoor and outdoor air pollution play a role in the current epidemic of pediatric asthma attacks. The infection causes more than half of all acute exacerbations of bronchial asthma in young children (20–23).

The present study found a significant decline in children with acute exacerbations of bronchial asthma after the COVID-19 pandemic. Large declines in visits for childhood asthma exacerbations after the pandemic were noted in the northeastern United States (24) and the Netherlands (25). The number of children hospitalized for bronchial asthma in the Slovenian region has also decreased by 70% compared to the same period during the last 3 years (26). On the one hand, this finding could be attributed to lifestyle changes following the COVID-19 outbreak. Following the outbreak, children in original childcare facilities, school environments, and home care were instructed to adapt to home network course learning, reduce gathering in public places, wash their hands frequently in daily life, disinfect contact items regularly, and wear a face mask. To some extent, these measures will aid in preventing or reducing respiratory infectious diseases. The discovery, on the other hand, could be linked to changes in meteorological and environmental factors following the COVID-19 pandemic.

Our research showed that the meteorological factors of monthly average temperature, humidity, rainfall, light speed, and wind speed did not change significantly before and after the pandemic. In contrast, the environmental factors NO_2_, SO_2_, PM_2.5_, and PM_10_ decreased after the pandemic when compared with those before the pandemic, whereas O_3_ levels increased. This finding is consistent with the result that the levels of all air pollutants decreased after the pandemic in Central São Paulo (27), and only O_3_ levels increased. During the most stringent level I response period in the Yangtze River Delta region of China, the levels of the major pollutants SO_2_, NOx, PM_2.5_, and VOCs decreased by 26, 47, 46, and 57%, respectively (28), and ozone concentration increased by 32.9%. A large number of studies have demonstrated that air pollution affects the respiratory system through pulmonary inflammation and oxidative stress, leading to short-term and long-term effects of obstructive respiratory diseases (asthma and chronic obstructive pulmonary disease [COPD]) and restrictive parenchymal lung diseases (fibrosis) (29, 30). Children exposed to high concentrations of NO_2_ are more likely to suffer from respiratory diseases, especially virus-induced asthma (23).

In our study, the increase in O_3_ levels is attributed to a significant reduction in NOx emissions during transportation, resulting in lower ozone titration (31). PM_2.5_ reduces atmospheric visibility and significantly blocks ultraviolet rays, causing ozone to rise. Some studies have found that ozone may have antiviral activity when exposed to it concurrently with virus infection (32). However, ozone must be available in high concentrations to reduce the airborne viral suspension, which is not the case at current environmental levels. Therefore, we expect that the overall effect of ozone will not reduce viral loads in humans or animals but rather weaken immune responses.

We also conducted a partial correlation analysis between acute asthma attack rate and environmental factors before and after the pandemic and found a positive correlation with PM_2.5_ after the pandemic but not before the pandemic. This could imply that when environmental pollution is reduced, and pollution indicators are reduced, PM_2.5_ has the strongest correlation with acute exacerbation of bronchial asthma. A 222-child study found that children living in areas with higher levels of PM_2.5_ had significantly lower levels of salivary lectins, one of the main antimicrobial proteins and peptides (33). A study conducted in Hefei, China, showed a significant short-term association between ambient PM_2.5_ levels and increased pneumonia-related hospitalizations (34). PM_2.5_ emitted by vehicles and industries increases the risk of hospitalization for asthma (35, 36). This could be because common pathogens that cause acute exacerbations of bronchial asthma to attach to PM-suspended particles and enter children's respiratory systems (37). Additionally, PM_2.5_ exposure can regulate viral attachment and entry into the body by altering the expression of viral receptors. Studies have shown that, 2 days after exposure to PM_2.5_, the expression level of ACE2 in the lungs of mice increases, suggesting that exposure to PM may alter viral entry by increasing the level of ACE2 of the SARS-CoV-2 receptor (38). Exposure to PM can also tilt the adaptive immune response toward an allergic response. *In vivo* experiments have shown that exposure to PM can promote allergic respiratory inflammation, enhance TH2 and TH17 responses, and increase the production of allergy-related IgE antibodies (39). Consistent with our results, an experiment performed at the University of California showed that PM_2.5_ exposure was associated with a higher chance of hospitalization only in patients with asthma or COPD (40).

The present study found a decrease in the prevalence of RSV, a common pathogen causing asthma, and an increase in the prevalence of rhinoviruses. The decline in RSV infections is most likely linked to lower levels of PM_2.5_ in the air. Studies at the individual level have shown a certain correlation between PM_2.5_ exposure and the incidence of respiratory infections. In children, both long-term and short-term exposures to PM_2.5_ are associated with hospitalization for RSV bronchiolitis, particularly in infancy (41). An experimental study found that when RSV was attached to ultrafine particles and then incubated *in vitro* with epithelial cells, the inflammatory response was greater than when RSV was used alone (42). The presence of fine particles can enhance viral infection and entry into the body in the case of RSV (43). Mice exposed to ultrafine particles emitted by combustion sources had a significantly reduced immune response and lower levels of protective cytokines such as tumor necrosis factor-α, lymphocytes, and interferons in bronchoalveolar lavage fluid. They increased inflammation and infection severity after RSV infection. HRV is another pathogen linked to asthma exacerbations and asthmatic respiratory infections in children. During the COVID-19 outbreak in Hong Kong (44), a large number of rhinoviruses were still detected in viral biopositive specimens, a finding that was consistent with our results. Liang et al. (45) showed that the use of surgical masks could significantly reduce influenza transmission through droplet infection but not HRV transmission. MP infection rate did not change before and after the pandemic.

After the pandemic, the incidence of acute bronchial asthma in school-age and adolescent children increased. This may be because, after the pandemic, kindergartens remained closed for a long time and had turned to home care, which reduced cross-infection in schools. Moreover, children were accompanied by guardians to avoid exposure to adverse outdoor environments such as sudden temperature changes. School-age children have more opportunities for outside exposure (46) and are exposed to large temperature changes. We discovered that the number of children with severe symptoms such as shortness of breath and dyspnea decreased, which could be due to the fact that the symptoms of acute asthma exacerbation are similar to those of COVID-19, and people are highly motivated to seek medical attention. Early detection and treatment can reduce the risk of complications caused by delayed treatment. Furthermore, an online questionnaire survey (47, 48) suggested that medication adherence was better during COVID-19 (49). Therefore, improved adherence may result in better asthma control and fewer hospitalizations.

Our study found that the incidence of acute asthma attacks before and after the pandemic was negatively correlated with monthly average temperature and O_3_. The multiple time series analysis showed that the ARIMA model based on temperature had the highest R^2^ value and was the most significant. Therefore, we established a temperature-based prediction model with a good degree of fit. A study conducted in Shanghai, China, found that lower temperatures were associated with an increase in asthma hospitalizations with a delay of 0–30 days (50), suggesting that cold weather is dangerous for children suffering from wheezing. Most studies have found that cold weather significantly impacts asthma visits or hospital admissions (51, 52). The body's immune response may be reduced in cold environments, reducing the body's ability to fight or kill pathogens and increasing adrenaline production. Furthermore, a drop in temperature stimulates the parasympathetic nervous system to produce mediators such as cysteine leukotrienes, resulting in increased bronchial smooth muscle contraction. Transient receptor potential melastatin 8 (TRPM8) is involved in the mechanism of cold temperature activation. TRPM8 has been studied as a potential trigger for pediatric asthma exacerbations. The TRPM8 receptor is involved in cold-induced mucus hypersecretion through the Ca(2+)-PLC-PIP2-Marcks signaling pathway (53). Fisher showed that TRPM8 is a key molecule responsible for respiratory sensations such as dyspnea, cold-induced asthma, and cough (54). Exposure to cold air may trigger airway obstruction and neutrophil inflammation in the lungs (55). Cold-dry air stimulation-induced small airway constriction has been observed in children with asthma (56, 57). All the above findings indicated that the pandemic did not affect monthly mean temperature and could predict the incidence of acute asthma attacks.

This study, however, has some limitations. The generalizability of our findings is limited due to the specific research field. Because of the obvious spatial heterogeneity of temperature bands, different results may be obtained. Therefore, we should apply our findings to other regions or populations our results to other regions or populations should be done with caution. Only inpatient children were counted; mild asthma exacerbations treated in the outpatient or emergency department were excluded from the study. With a retrospective design, we relied on discharge codes to identify target subjects, which may have excluded some potential subjects due to errors in the discharge input process. We only looked at the relationship between ambient temperature and more severe asthma exacerbations that required hospitalization, which may have overestimated the effect of temperature variations. Finally, due to statistical method limitations, we have provided limited evidence on the relationship between meteorological or environmental factors and the development of asthma disease. More sophisticated models, such as Bayesian statistics and distributed lag non-linear models, are required to provide more evidence on environmental epidemiology and improve predictions.

Despite this, the source of bronchial asthma in our hospital accounted for at least 90% of the local area, and our results well reflect the population-based estimate for hospitalization. Moreover, we included a large number of patients to eliminate information deviation, and we used multiple linear regression analysis to determine the meteorological and environmental risk factors for bronchial asthma and the influence of the rate of bronchial asthma. The findings of this study are useful and critical for predicting asthma attack rates.

## Data availability statement

The original contributions presented in the study are included in the article/supplementary material, further inquiries can be directed to the corresponding authors.

## Ethics statement

The studies involving human participants were reviewed and approved by the Institutional Human Ethical Committee of Children's Hospital of Soochow University with judgment's reference number 2020CS078. Written informed consent to participate in this study was provided by the participants' legal guardian/next of kin. Written informed consent was obtained from the individual(s), and minor(s)' legal guardian/next of kin, for the publication of any potentially identifiable images or data included in this article.

## Author contributions

SG and ZC conceived and designed the study. LH, DC, and JC made contributions to the analysis and interpretation of data. CZ collected the clinical data. All authors have read and approved the manuscript.

## References

[1] Wang C Horby PW Hayden FG Gao GF. A novel coronavirus outbreak of global health concern. Lancet. (2020) 395:470–3. 10.1016/S0140-6736(20)30185-9 31986257 PMC7135038

[2] Deng S-Q Peng H-J. Characteristics of and public health responses to the coronavirus disease 2019 outbreak in China. J Clin Med. (2020) 9:575. 10.3390/jcm9020575 32093211 PMC7074453

[3] Chen K Wang M Huang C Kinney PL Anastas PT. Air pollution reduction and mortality benefit during the COVID-19 outbreak in China. Lancet Planetary Health. (2020). 10.1101/2020.03.23.20039842 32411944 PMC7220178

[4] Wang Y Yuan Y Wang Q Liu C Zhi Q Cao J. Changes in air quality related to the control of coronavirus in China: implications for traffic and industrial emissions. Sci Total Environ. (2020) 731:139133. 10.1016/j.scitotenv.2020.139133 32402905 PMC7202850

[5] Zhang Y Xiang Q Yu C Yang Z. Asthma mortality is triggered by short-term expos ures to ambient air pollutants: Evidence from a Chinese urban population. Atmosphe ric Environ. (2020) 223:117271–80. 10.1016/j.atmosenv.2020.117271

[6] Johansson H Mersha TB Brandt EB Hershey GK. Interactions between environmental pollutants and genetic susceptibility in asthma risk. Current Opin Immunol. (2019) 60:156–162. 10.1016/j.coi.2019.07.010 31470287 PMC6800636

[7] Wang J Zhang Y Li B Zhao Z Huang C Zhang X. Asthma and allergic rhinitis among young parents i n China in relation to outdoor air pollution, climate and home environment. Sci Total Environ. (2021) 751:141734–43. 10.1016/j.scitotenv.2020.141734 32882555

[8] Perez L Declercq C Iniguez C Aguilera I Badaloni C Ballester F. Chronic burden of near-roadway traffic pollution in 10 European cities (APHEKOM network). Eur Respir J. (2013) 42:594–605. 10.1183/09031936.00031112 23520318

[9] Romaszko-Wojtowicz A Cymes I Dragańska E Doboszyńska A Romaszko J Glińska-Lewczuk K. Relationship between biometeor ological factors and the number of hospitalizations due to asthma. Sci Rep. (2020) 10:9593–601. 10.1038/s41598-020-66746-8 32533079 PMC7293260

[10] Shu W. A meta-analysis of the prevalence of childhood asthma in China from 2011 to 2018. Chinese School Health. (2020) 41:94–97. 10.16835/j.cnki.1000-9817.2020.08.023

[11] Editorial Editorial Committee of Chinese Journal of Pediatrics, Respiratory Respiratory Group of Chinese Pediatrics Society, Chinese Medical Doctor Association, Pediatric Pediatric Respiratory Committee of Pediatricians Society. Recommendations for standardized diagnosis and treatment of bronchial asthma in children. Chin J Pediatrics. (2020) 58:708–17. 10.3760/cma.j.cn112140-20200604-00578 32872710

[12] Respiratory Group, Pediatrics Pediatrics Branch of Chinese Medical Association, Editorial Editorial Board of the Chinese Journal of Pediatrics. Guidelines for the diagnosis and Treatment of bronchial asthma in children. Chin J Pediatrics. (2016) 54:167–81. 10.3760/ema.j.issn.0578-1310.2016.03.003

[13] The Global Strategy, W. H. O. for Women's, Children's and Adolescents' Health (2016–2030) [EB/OL]. (2018). Available online at: https://www.who.int/publication/i/item/the-Global-Strategy-for-Women-s-Children-s-and-Adolescents-Health(2016-2030)-early-childhood-development-report-by-the-director-general (accessed November 30, 2021).

[14] Liu T-T Qi J-L Yin J Gao Q Xu W Qiao J-J. Asthma mortality among children and adolescentsin China, 2008–2018. World J Pediatr. (2022) 10:1–9. 10.1007/s12519-022-00548-y 35536454

[15] Huang K Yang T Xu J Yang L Zhao J Zhang X. Prevalence, risk factors, and management of asthma in China: a national cross-sectional study. Lancet. (2019) 394:407–18. 10.1016/S0140-6736(19)31147-X 31230828

[16] Eguiluz-Gracia I Mathioudakis AG Bartel S Vijverberg SJH Fuertes E Comberiati P. The need for clean air: the way air pollution and climate change affect allergic rhinitis and asthma. Allergy. (2020) 75:2170–84. 10.1111/all.14177 31916265

[17] Hu Y Xu Z Jiang F Li S Liu S Wu M. Relative impact of meteorological factors and air pollutants on childhood allergic diseases in Shanghai, China. Sci Total Environ. (2020) 706:135975. 10.1016/j.scitotenv.2019.135975 31841850

[18] Beasley R Semprini A Mitchell EA. Risk factors for asthma: is prevention possible? Lancet. (2015) 386:1075–85. 10.1016/S0140-6736(15)00156-7 26382999

[19] Ducharme FM Tse SM Chauhan B. Diagnosis, management, and prognosis of pre-school wheeze. Lancet. (2014) 383:1593–604. 10.1016/S0140-6736(14)60615-2 24792856

[20] Kuitunen I Artama M Mäkelä L Backman K Heiskanen-Kosma T Renko M. Effect of social distancing due to the COVID-19 pandemic on the incidence of viral respiratory tract infections in children in Finland during early 2020. Pediatr Infect Dis J. (2020) 39:e423–7. 10.1097/INF.0000000000002845 32773660

[21] Trenholme A Webb R Lawrence S Arrol S Taylor S Ameratunga S. COVID-19 and infant hospitalizations for seasonal respiratory virus infections, New Zealand, 2020. Emerg Infect Dis. (2021) 27:641–3. 10.3201/eid2702.204041 33263515 PMC7853573

[22] Huang QS Wood T Jelley L Jennings T Jefferies S Daniells K. Impact of the COVID-19 non-pharmaceutical interventions on influenza and other respiratory viral infections in New Zealand. Nat Commun. (2021) 12:1–7. 10.1038/s41467-021-21157-9 33579926 PMC7881137

[23] Han A Deng S Yu J Zhang Y Jalaludin B Huang C. Asthma triggered by extreme temperatures: from epidemiological evidence to biological plausibility. Environ Res. (2022) 216:114489. 10.1016/j.envres.2022.114489 36208788

[24] Kenyon CC Hill DA Henrickson SE Bryant-Stephens TC Zorc JJ. Initial effects of the COVID-19 pandemic on pediatric asthma emergency department utilization. J Allergy Clin Immunol Pract. (2020) 8:2774–6. 10.1016/j.jaip.2020.05.045 32522565 PMC7483361

[25] Kruizinga MD Peeters D van Veen M. The impact of lockdown on pediatric ED visits and hospital admissions during the COVID19 pandemic: a multicenter analysis and review of the literature. Eur J Pediatr. (2021) 180:2271–9. 10.1007/s00431-021-04015-0 33723971 PMC7959585

[26] Krivec U Seliger AK Tursic J. COVID-19 lockdown dropped the rate of paediatric asthma admissions. Arch Dis Child. (2020) 105:809–10. 10.1136/archdischild-2020-319522 32444452 PMC7392481

[27] Nakada LYK Urban RC. COVID-19 pandemic: impacts on the air quality during the partial lockdown in São Paulo state. Brazil Sci Total Environ. (2020) 730:139087. 10.1016/j.scitotenv.2020.139087 32380370 PMC7189200

[28] Li Q Guan X Wu P Wang X Zhou L Tong Y. Early transmission dynamics in Wuhan, China, of novel coronavirus infected pneumonia. N Engl J Med. (2020) 382:1199–207. 31995857 10.1056/NEJMoa2001316 PMC7121484

[29] Garcia E Berhane KT Islam T McConnell R Urman R Chen Z. Association of changes in air quality with incident asthma in children in California, 1993–2014. JAMA. (2019) 321:1906–15. 10.1001/jama.2019.5357 31112259 PMC6537847

[30] Liang L Cai Y Barratt B Lyu B Chan Q Hansell AL. Associations between daily air quality and hospitalisations for acute exacerbation of chronic obstructive pulmonary disease in Beijing, 2013–17: an ecological analysis. Lancet Planet Health. (2019) 3:e270–9. 10.1016/S2542-5196(19)30085-3 31229002 PMC6610933

[31] Sicard P De Marco A Agathokleous E Feng Z Xu X Paoletti E. Amplified ozone pollution in cities during the COVID-19 lockdown. Sci Total Environ. (2020) 735:139542. 10.1016/j.scitotenv.2020.139542 32447070 PMC7237366

[32] Dubuis ME Dumont-Leblond N Laliberté C. Ozone efficacy for the control of airborne viruses:bateriophage and norovirus models. PLoS ONE. (2020) 15:e0231164. 10.1371/journal.pone.0231164 32275685 PMC7147755

[33] Zhang S Huo X Zhang Y Huang Y Zheng X Xu X. Ambient fine particulate matter inhibits innate airway antimicrobial activity in pre-school children in e-waste areas. Environ Int. (2019) 123:535–42. 10.1016/j.envint.2018.12.061 30622078

[34] Yan S Wang X Yao Z Cheng J Ni H Xu Z. Seasonal characteristics of temperature variability impacts on childhood asthma hospitalization in Hefei, China: If PM2, 5. modify the association?. Environ Res. (2021) 207:112078. 10.1016/j.envres.2021.112078 34599899

[35] Glencross DA Ho TR Camilla N Hawrylowicz CM Pfeffer PE. Air pollution and its effects on the immune system. Free Radic Biol Med. (2020) 151:56–68. 10.1016/j.freeradbiomed.2020.01.179 32007522

[36] Sarmadi M Marufi N Kazemi Moghaddam V. Association of COVID-19 global distribution and environmental and demographic factors: an updated three-month study. Environ Res. (2020) 188:109748. 10.1016/j.envres.2020.109748 32516636 PMC7258807

[37] Chen PS Tsai FT Lin CK Yang CY Chan CC Young CY. Ambient influenza and avian influenza virus during dust storm days and background days. Environ Health Perspect. (2010) 118:1211–6. 10.1289/ehp.0901782 20435545 PMC2944079

[38] Mendy A Wu X Keller JL Fassler CS Apewokin S Mersha TB. Air pollution and the pandemic: Long-term PM2.5 exposure and disease severity in COVID-19 patients. Respirology. (2021) 26:1181–7. 10.1111/resp.14140 34459069 PMC8662216

[39] Gilmour MI. Influence of air pollutants on allergic sensitization: the paradox of increased allergies and decreased resistance to infection. Toxicol Pathol. (2012) 40:312–4. 10.1177/0192623311431949 22222885

[40] Mendy A Wu X Keller JL Fassler CS Apewokin S Mersha TB. Long-term exposure to fine particulate matter and hospitalization in COVID-19 patients. Respir Med. (2021) 178:106313. 10.1016/j.rmed.2021.106313 33550152 PMC7835077

[41] Horne BD Joy EA Hofmann MG Gesteland PH Cannon JB Lefler JS. Short-term elevation of fine particulate matter air pollution and acute lower respiratory infection. Am J Respir Crit Care Med. (2018) 198:759–66. 10.1164/rccm.201709-1883OC 29652174

[42] Cruz-Sanchez TM Haddrell AE Hackett TL. Formation of a stable mimic of ambient particulate matter containing viable infectious respiratory syncytial virus and its dry-deposition directly onto cell cultures. Anal Chem. (2013) 85:898–906. 10.1021/ac302174y 23205519

[43] Groulx N Urch B Duchaine C Mubareka S Scott JA. The pollution particulate concentrator (PoPCon): a platform to investigate the effects of particulate air pollutants on viral infectivity. Sci Total Environ. (2018) 628:1101–7. 10.1016/j.scitotenv.2018.02.118 30045533

[44] Wong KL Wong WHS Yau YS Lee SL Chiu SSS. Asthma admission among children in Hong Kong during the first year of the COVID-19 pandemic. Pediatr Pulmonol. (2022) 1–7. 10.1002/ppul.26141 36097891

[45] Leung NH Chu DK Shiu EY Chan KH McDevitt JJ Hau BJ. Respiratory virus shedding in exhaled breath and efficacy of face masks. Nature Med. (2020) 26:676–80. 10.1038/s41591-020-0843-2 32371934 PMC8238571

[46] Kim H Kim H Lee JT. Assessing the cold temperature effect on hospital visit by allergic rhinitis in Seoul, Korea. Sci Total Environ. (2018) 633:938–45. 10.1016/j.scitotenv.2018.03.166 29758916

[47] Chavasse RJ. Covid-19: reduced asthma presentations in children. BMJ. (2020) 370:m2806. 10.1136/bmj.m2806 32669279

[48] Kaye L Theye B Smeenk I Gondalia R Barrett MA Stempel DA. Changes in medication adherence among patients with asthma and COPD during the COVID-19 pandemic. J Allergy Clin Immunol Pract. (2020) 8:2384–5. 10.1016/j.jaip.2020.04.053 32371047 PMC7194036

[49] Papadopoulos NG Custovic A Deschildre A. Impact of COVID-19 on pediatric asthma: practice adjustments and disease burden. J Allergy Clin Immunol Pract. (2020) 8:2592–9. 10.1016/j.jaip.2020.06.001 32561497 PMC7297686

[50] Zhou Y Pan J Xu R Lu W Wang Y Liu T. Asthma mortality attributable to ambient temperatures: a case-crossover study in China. Environ Res. (2022) 214:114116. 10.1016/j.envres.2022.114116 35988831

[51] Kabir AF Ng CFS Yasumoto S Hayashi T Watanabe C. Effect of ambient temperature on daily nebulized asthma hospital visits in a tropical city of Dhaka, Bangladesh. Int J Environ Res Public Health. (2021) 18:890. 10.3390/ijerph18030890 33498592 PMC7908622

[52] Cong X Xu X Zhang Y Wang Q Xu L Huo X. Temperature drop and the risk of asthma: a systematic review and meta-analysis. Environ Sci Pollut Res Int. (2017) 24:22535–46. 10.1007/s11356-017-9914-4 28804860

[53] Li M Li Q Yang G Kolosov VP Perelman JM Zhou XD. Cold temperature induces mucin hypersecretion from normal human bronchial epithelial cells *in vitro* through a transient receptor potential melastatin 8 (TRPM8)-mediated mechanism. J Allergy Clin Immunol. (2011) 128:626–634. 10.1016/j.jaci.2011.04.032 21762971

[54] Li S Baker PJ Jalaludin BB Marks GB Denison LS Williams GM. Ambient temperature and lung function in children with asthma in Australia. Eur Respir J. (2014) 43:1059–66. 10.1183/09031936.00079313 24311765

[55] Kennedy MD Steele AR Parent EC Steinback CD. Cold air exercise screening for exercise induced bronchoconstriction in cold weather athletes. Respir Physiol Neurobiol. (2019) 269:103262. 10.1016/j.resp.2019.103262 31369875

[56] Steinbacher M Pfleger A Schwantzer G Jauk S Weinhandl E Eber E. Small airway function before and after cold dry air challenge in pediatric asthma patients during remission. Pediatr Pulmonol. (2017) 52:873–9. 10.1002/ppul.23724 28486753

[57] Schinasi LH Kenyon CC Hubbard RA Zhao Y Maltenfort M Melly SJ. Associations between high ambient temperatures and asthma exacerbation among children in Philadelphia, PA: a time series analysis. Occup Environ Med. (2022) 1–7:107823. 10.1136/oemed-2021-107823 35246484

